# Green Compressed Fluid Technologies To Extract Antioxidants and Lipids from *Galdieria phlegrea* in a Biorefinery Approach

**DOI:** 10.1021/acssuschemeng.9b07505

**Published:** 2020-01-31

**Authors:** Paola Imbimbo, Monica Bueno, Luigi D’Elia, Antonino Pollio, Elena Ibañez, Giuseppe Olivieri, Daria Maria Monti

**Affiliations:** † Department of Chemical Sciences, University of Naples Federico II, via Cinthia 4, 80126 Naples, Italy; ‡ Laboratory of Foodomics, Institute of Food Science Research, CIAL, CSIC, Nicolás Cabrera 9, 28049 Madrid, Spain; § Department of Biology, University of Naples Federico II, via Cinthia 4, 80126 Naples, Italy; ∥ Bioprocess Engineering Group, Wageningen University and Research, Droevendaalsesteeg 1, 6700AA Wageningen, The Netherlands; ⊥ Department of Chemical, Materials and Industrial Engineering, University of Naples Federico II, Piazzale Tecchio 80, 80125 Naples, Napoli, Italy

**Keywords:** phycocyanin, carotenoids, lipids, compressed fluid technologies, biorefinery, microalgae, *Galdieria phlegrea*

## Abstract

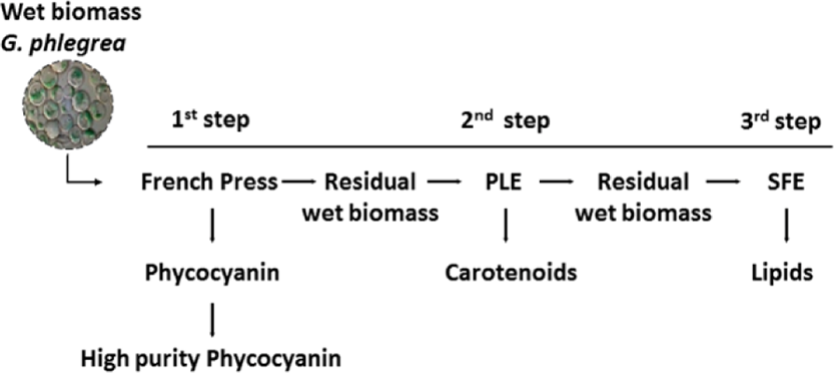

A green cascade approach was used to recover phycocyanins, carotenoids and lipids from *Galdiera phlegrea*. Phycocyanin extraction was performed by high pressure homogenization and purified by ultrafiltration, whereas carotenoids were obtained by a pressurized liquid extraction and lipids by supercritical fluid extraction. The second step of this innovative, green, and cost-effective procedure is able to improve the recovery of zeaxanthin and β-carotene up to 40%, without affecting the quality of compounds and avoiding the use of organic solvents and the drying processes. The isolated carotenoids were active as antioxidants, as clearly shown by their protective activity on a cell-based model. The lipid yield was increased by 12% with respect to conventional methods.

## Abstract 2

Compressed fluid extractions allow the recovery of high-value molecules from *Galdiera phlegrea* by using a green biorefinery approach.

## Introduction

Microalgae are a continuous and reliable source of safe natural and high-value products, such as soluble proteins, polyunsaturated fatty acids, and pigments. Phycocyanins (PCs) are blue colored, highly fluorescent, and water-soluble proteins, synthetized in cyanobacteria and red algae. PCs, as the other phycobiliproteins, are antenna pigments that can improve the photosynthetic efficiency of microalgae. Because of their brilliant color, PCs are commonly used in cosmetic and food industry.^1^ They are also endowed with therapeutic properties such as antioxidant, anti-inflammatory, hepato-protective, and antitumoral activity.^2^ Among pigments, carotenoids function as accessory pigments in a light-harvesting photosystem during photosynthesis,^3^ and they are also important for their antioxidant function, as they deactivate free radicals, thus preventing cell damages. In the last decades, carotenoids have attracted great interest because of their beneficial effect on human health. The demand of carotenoids is rapidly growing: the global carotenoid market was estimated to be ∼1.24 billion USD in 2016 and is projected to increase to ∼1.53 billion USD by 2021, at a compound annual growth rate of 3.78% from 2016 to 2021.^4^ To date, commercially available carotenoids are generally synthetic because they are more stable than natural ones as they are formulated to minimize oxidation or isomerization.^5^ However, the emulsified preparations of synthetic carotenoids show high toxicity, carcinogenicity, and teratogenicity properties, thus generating criticism among health-conscious consumers.^5,6^ With microalgae being good producers of many pigments, the extraction of carotenoids from these microorganisms would be very competitive in the market and would have a huge economic impact.^7^ Microalgae can accumulate also significant amount of lipids (from 1 to 70%),^8^ depending on the strain and the culture conditions.^9^ Lipids can be employed as feedstock for nutraceutical, pharmaceutical, foods, and biofuel industries. To date, the bioenergy market has the lowest value. This is due to the fact that biogas, bioethanol, and biodiesel have a selling price of 0.2 € m^–3^, 0.4 € kg^–1^, and 0.5 € L^–1^ respectively, a price that still exceeds their high downstream process costs (20.5 € m^–3^, 33.34 € kg^–1^, and 25.56 g € L^–1^, respectively).^10^ Thus, an improvement in efficient, cost-effective, and green extraction techniques to produce high-quality compounds is needed. In this context, microalgae are an excellent source of molecules endowed with biological activity. Notably, the design of a suitable integrated biorefinery platform can efficiently extract target compounds in a cascade approach and, in accordance with the green chemistry principles, is still a challenge. Among all the innovative techniques, compressed fluid extractions are considered the most competitive ones because they may fulfill this criteria.^11^ In this context, pressurized liquid extraction (PLE) and supercritical fluid extraction (SFE) are the most widely employed as they could be based on the use of the same system; so they would represent process intensification. PLE and SFE are innovative techniques that use pressurized solvents at an elevated temperature and pressure to extract molecules. Moreover, the extraction performance is enhanced as compared to those techniques carried out at near room temperature and atmospheric pressure.^11−13^

**Figure 1:**
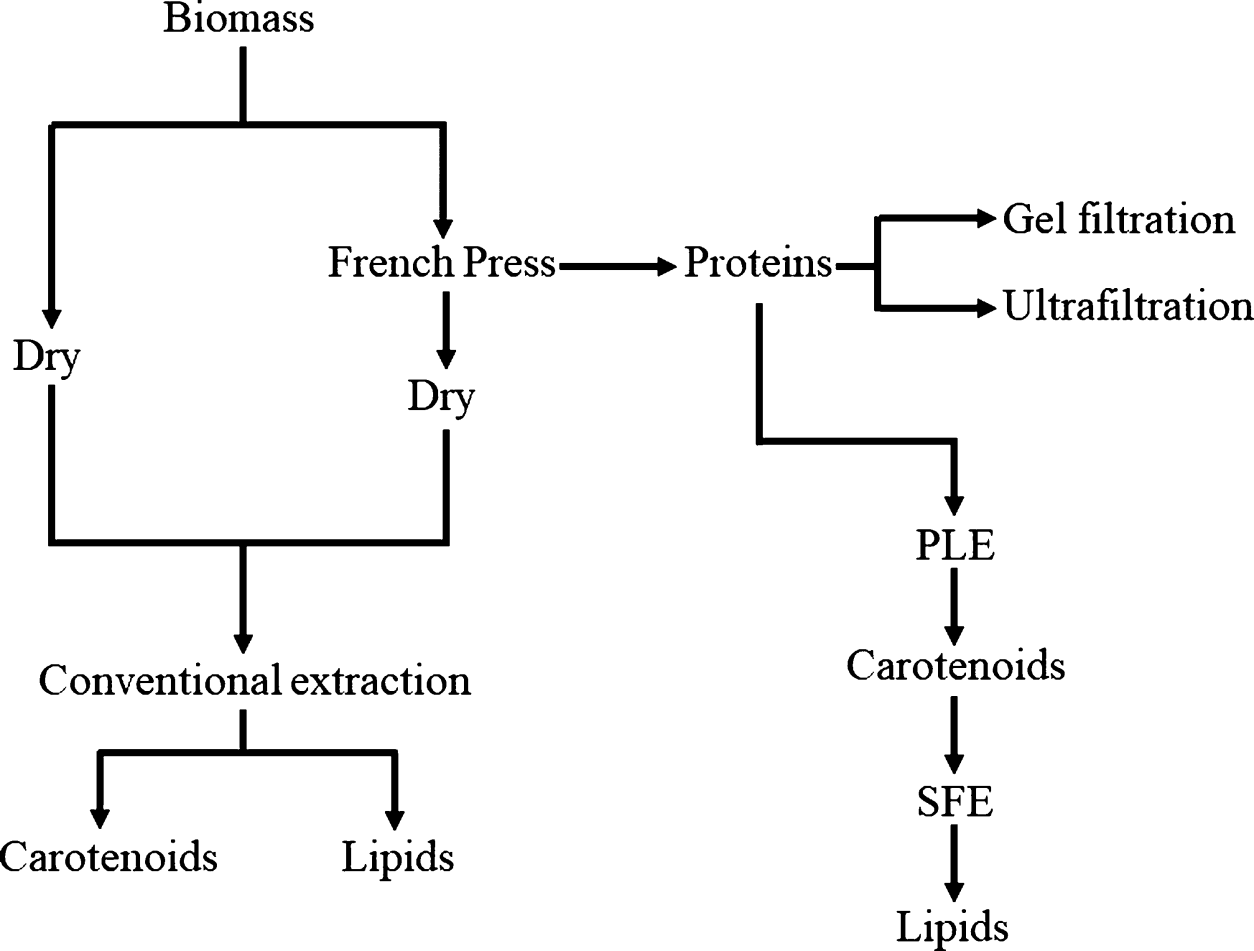
Schematic representation of the extraction strategy.

In the present work, we set up a cascade approach to recover high value bioproducts from *Galdieria phlegrea*, a unicellular thermo-acidophilic red alga. The experimental strategy is reported in Figure 1. Starting from the previously established technique used to disrupt cells and extract PCs,^14^ an optimization of the isolation of PC was carried out. Then, the residual wet biomass was used to extract two different bioproducts in two sequential steps: carotenoids by using PLE and finally lipids by SFE. The bioactivity of the extracted carotenoids obtained by PLE was validated on a cell-based model, using human immortalized keratinocytes and compared to the bioactivity of the commercial molecules.

## Materials and Methods

### Reagents

High performance liquid chromatography (HPLC)-grade acetone and methanol were from VWR (Barcelona, Spain). Antibodies were from Cell Signal Technology (Danvers, MA, USA). All the other reagents and standards were from Sigma-Aldrich (Madrid, Spain).

### Microalgal Strain and Culture Conditions

*G. phlegrea* (strain 009) was provided from the Algal Collection of the University Federico II (ACUF, http://www.acuf.net). Cells were grown in autotrophic conditions in photobioreactors, as described in Imbimbo et al.^14^

### PC Extraction and Purification

After harvesting the biomass by centrifugation at 1200*g* for 30 min at room temperature, cells were suspended in 50 mM sodium acetate pH 5.5.^15^ Cell disruption was performed by high-pressure (French Press). Two consecutive cycles, each at 2 kbar, were performed to disrupt the biomass. The cell lysate was obtained by centrifugation at 5000*g* at 4 °C for 30 min, and proteins were recovered in the supernatant, whereas the residual biomass was used for further extractions. To purify PC, two single step purification techniques were used in parallel: gel-filtration and ultrafiltration.

The size-exclusion chromatography was performed by using a Sephadex G-75 fine equilibrated in 50 mM sodium acetate pH 5.5. The ultrafiltration was performed by using 100 kDa molecular weight cut off membranes, and the process was performed at room temperature. At the end of the purification, the permeate was discarded and the retentate was collected. The grade of purity of PC was calculated by measuring the ratio *A*_620nm_/*A*_280nm_.

### Storage of Biomass

The residual wet biomass, after protein extraction, was stored at −80 °C. To avoid that the storage conditions would affect the results, extraction of carotenoids was performed after 72 h.

### Conventional Carotenoid Extraction

Carotenoids were extracted using the method of Reyes et al.^16^ Briefly, 200 mg of lyophilized biomass was mixed with 20 mL of HPLC-grade acetone containing 0.1% (w/v) butylate hydroxytoluene, and the mixture was shaken for 24 h in a thermostatic shaker at 500 rpm and 20 °C. Then, the sample was centrifuged at 4 °C for 10 min at 5000*g*. The supernatant was collected, and the solvent was removed under N_2_ stream. The extracts were weighted and stored in the dark at −20 °C.

### Conventional Lipid Extraction

Total lipid extraction was performed according to the Axelsson and Gentili method.^17^ Freeze-dried microalgae biomass (25 mg) was mixed with 8 mL of chloroform/methanol 2:1 (v/v). Then, 2 mL of NaCl 0.73% (w/v) was added and mixed again. The sample was centrifuged at 350*g* for 5 min at room temperature, allowing the separation of the two phases. The lower layer was removed and collected. The solvent was removed under N_2_ stream. The extracts were weighted and stored in the dark at −20 °C.

### Compressed Fluid Extraction Processes

All high pressure extractions were performed in a homemade compressed fluid extractor coupled to a PU-2080 HPLC pump from Jasco (Tokyo, Japan). This equipment can be employed to carry out both PLE and SFE. To this purpose, 2 g of wet algal biomass (the equivalent of 200 mg of dried biomass) were mixed with silica gel of 150 Å (*S*150) pore size with a particle size of 200–425 mesh. The required amount of this silica gel was added as an adsorbent till a static paste was obtained.^18^ Silica prevents the paste draining in the equipment pipeline when loading in the extraction cell and improves the solute recovery.^18^ The mixture was added into a stainless-steel extraction cell sandwiched between glass wool to prevent clogging problems. Extractions were carried out in triplicate in two sequential steps, decreasing the polarity of the solvents, in order to exhaust the microalgae biomass of relevant extractable compounds. PLE was performed at a static extraction mode at 100 bar, 50 °C for 30 min using pure ethanol as a solvent. The extracts were collected in glass vials, dried under N_2_ stream, and then weighed and stored at −20 °C in the dark. Subsequently, the residue of the previous extraction was used as a raw material for the next step. SFE was carried out in the same apparatus, using pure CO_2_ as a solvent. The extraction was performed at 350 bar, 60 °C for 100 min. The CO_2_ flow rate was set up at 5 mL/min. Pressure was controlled by using a back pressure regulator. The extracts were collected in glass vials, dried under N_2_ stream, and then weighed and stored at −20 °C in the dark. A schematic representation of the used apparatus is reported in Figure S1 (Supporting Information).

### Total Carotenoid Determination

The total carotenoid content was determined spectrophotometrically as described by Gilbert-López et al.^19^ The extracts from PLE were dissolved in pure methanol in a concentration ranging from 0.05 to 5 mg/mL. A standard calibration curve of β-carotene (from 5 to 200 μg/mL) was used to calculate the concentration of total carotenoids. The absorbance of samples was recorded at 470 nm. The total carotenoid content was expressed as the ratio of mg of carotenoids and g of the extract. The carotenoid yield was expressed as mg of carotenoids extracted per g of dry biomass. Analyses was carried out in triplicate.

### Carotenoid Characterization by HPLC–DAD–MS

Carotenoids were characterized by HPLC–DAD using the method described by Castro-Puyana et al.,^20^ with some modifications. HPLC analyses were performed using an Agilent 1100 series liquid chromatograph (Santa Clara, CA, USA) equipped with a diode-array detector and using a YMC-C_30_ reversed-phase column (250 mm × 4.6 mm inner diameter, 5 μm particle size; YMC Europe, Schermbeck, Germany) and a precolumn YMC-C_30_ (10 mm × 4 mm i.d., 5 μm). The mobile phase was a mixture of methanol–MTBE–water (90:7:3, v/v/v) (solvent A) and methanol–MTBE (10:90, v/v) (solvent B). Carotenoids were eluted according to the following gradient: 0 min, 0% B; 20 min, 30% B; 35 min, 50% B; 45 min, 80% B; 50 min, 100% B; 60 min, 100% B; 62 min, 0% B. The flow rate was 0.8 mL/min while the injection volume was 10 μL. The detection was performed at 280, 450, and 660 nm, although the spectra from 240 to 770 nm were recorded using the DAD (peak width >0.1 min (2 s) and slit 4 nm). The instrument was controlled by LC Chem Station 3D Software Rev. B.04.03 from Agilent. Extracts were dissolved in pure methanol in a concentration ranging from 1 to 10 mg/mL to 10 and filtered through 0.45 μm nylon filters before HPLC analysis. Each dilution was injected in triplicate. For calibration plots, different concentrations of zeaxanthin (from 3.9 to 62.5 μg/mL) and of β-carotene (from 31.3 to 1000 μg/mL) were analyzed in duplicate as described in Gallego et al.^21^ The same instrument was directly coupled at the exit of the DAD to an Agilent ion trap 6320 mass spectrometer (Agilent Technologies) via an atmospheric pressure chemical ionization interface. Analyses were conducted under the positive ionization mode using the parameters described elsewhere.^21^ This time extracts were dissolved in pure methanol in concentrations between 10 and 20 mg/mL and injected in duplicate. Automatic tandem mass spectrometry (MS/MS) analyses were also performed fragmenting the two highest precursor ions.

### ABTS Assay

The antioxidant activity of the lipophilic extract was evaluated by ABTS assay (2,2′-azinobis-[3-ethylbenzthiazoline-6-sulfonic acid]). The colorimetric assay is based on the reduction of the ABTS^+^ radical by the antioxidant molecules present in the sample. The radical is produced by the reaction of a 7 mM ABTS solution mixed with 2.45 mM of potassium persulfate conducted for 16 h at room temperature in the dark. The mixture is then diluted in deionized water to obtain an absorbance of 0.7 ± 0.02 at 734 nm. The lipophilic extract in different concentrations was allowed to react with ABTS for 7 min in the dark, and the absorbance was measured at 734 nm again. Trolox (6-hydroxy-2,5,7,8-tetramethylchromane-2-carboxylic acid) was used as a standard to obtain a calibration curve. Each extract was analyzed three times in triplicate.

### Cell Culture and Cytotoxicity Assay

Human immortalized keratinocytes (HaCaT) were from Innoprot (Biscay, Spain), whereas immortalized murine fibroblasts (BALB/c 3T3) were from ATCC (Manassas, Virginia). Cells were cultured in 10% fetal bovine serum in Dulbecco’s modified Eagle’s medium, in the presence of 1% antibiotics and 2 mM l-glutamine, in a 5% CO_2_ humidified atmosphere at 37 °C. HaCaT cells were seeded in 96-well plates at a density of 2 × 10^3^cells/well and BALB/c 3T3 at a density of 3 × 10^3^cells/well. Approximately 24 h after seeding, increasing concentrations of the lipophilic extract (from 10 to 100 μg/mL) were added to the cells for different lengths of time. At the end of each experimental point, cell viability was measured by the MTT assay, as described by Arciello et al.^22^ Cell survival was expressed as the percentage of viable cells in the presence of the lipophilic extract compared to control cells (represented by the average obtained between untreated cells and cells supplemented with the highest concentration of buffer). Each sample was tested in three independent analyses, each carried out in triplicates.

### DCFDA Assay

The antioxidant effect of the lipophilic extract (50 μg/mL) was measured by determining the intracellular ROS levels. The protocol used by Del Giudice et al. was followed,^23^ with some modifications. Briefly, HaCaT cells were exposed for different lengths of time to the extract under test and then irradiated by UVA light for 10 min (100 J/cm^2^). Fluorescence intensity of the fluorescent probe (2′,7′-dichlorofluorescein, DCF) was measured at an emission wavelength of 525 nm and an excitation wavelength of 488 nm using a Perkin-Elmer LS50 spectrofluorimeter (Shelton, CT, USA). Emission spectra were acquired at a scanning speed of 300 nm/min, with 5 slit width both for excitation and emission. ROS production was expressed as a percentage of DCF fluorescence intensity of the sample under test, compared to the untreated sample. Three independent experiments were carried out, each one with three determinations.

### Determination of Lipid Peroxidation Levels

The levels of lipid peroxidation were determined by using the thiobarbituric acid reactive substances (TBARS) assay according to the protocol proposed by Petruk et al.^24^ Briefly, HaCaT cells were preincubated for 15 and 30 min with the lipophilic extract and then irradiated by UVA light for 10 min (100 J/cm^2^). Cells were detached by trypsin and centrifuged at 1000*g* for 10 min, 5 × 10^5^ cells were resuspended in 0.67% thiobarbituric acid (TBA), and an equal volume of 20% trichloroacetic acid was added. Samples were then heated at 95 °C for 30 min, incubated on ice for 10 min, and centrifuged at 3000*g* for 5 min, at 4 °C. TBA reacts with the oxidative degradation products of lipids in samples, yielding red complexes that absorb at 532 nm. Lipid peroxidation levels were expressed as a percentage of absorbance at 532 nm of the sample under test, compared to the untreated sample. Three independent experiments were carried out, each one with three determinations.

### Western Blot Analysis

HaCaT cells were seeded at a density of 3 × 10^5^ cells/cm^2^ in a complete medium for 24 h and then treated with 50 μg/mL of the lipophilic extract for different lengths of time. To analyze Nrf-2 expression levels, nuclear and cytosolic lysates were prepared as follows. Cells were detached by trypsin and centrifuged at 1000*g* for 10 min. Pellets were resuspended in lysis buffer (0.5% Triton X-100 in PBS pH 7.4) containing protease and phosphate inhibitors. After 20 min incubation on ice, samples were centrifuged at 1200*g* for 5 min at 4 °C. The supernatants were removed and collected as cytosolic lysates. The residual pellets were washed in the same buffer and resuspended in RIPA buffer (50 mM Tris-HCl pH 7.4, 1% NP-40, 0.25% Na deoxycholate, 150 mM NaCl, 1 mM EDTA) completed with protease and phosphatase inhibitors. After 20 min incubation on ice, vortexing every 5 min, samples were centrifuged at 14,000*g* for 30 min at 4 °C. The supernatants were collected as nuclear lysates. The concentration of samples was determined by the Bradford assay, and the samples were analyzed by sodium dodecyl sulfate-polyacrylamide gel electrophoresis and western blot analysis. To normalize protein intensity levels, antibodies against β-actin and B23 were used. The chemiluminescence detection system was from Bio-Rad (Hercules, CA, USA).

### Statistical Analysis

Experimental data results were analyzed by ANOVA, and means were compared by Tukey’s HSD (SPSS statics V15 IBM, New York, United States). The value of *p* ≤ 0.05 was considered statistically significant, figured by alphabetical letters along means in tables.

## Results and Discussion

### Optimization of PC Purification

We recently set up a procedure to disrupt *G. phlegrea* biomass by using a conventional high-pressure procedure. PC was then easily recovered from the supernatant by a single purification step, that is, gel filtration.^14^ However, from an economic point of view, the size exclusion chromatography is not feasible, as it is difficult to be scaled-up. Thus, we optimized PC purification by using ultrafiltration and compared the results with those previously obtained. As shown in Table 1, both the ultrafiltration technique and gel filtration allow obtaining a PC with a high purity grade. It is known that a purity grade ≤0.7 is indicative of a food grade, between 0.7 and 3.9 is of reagent grade, and ≥4.0 of analytical grade.^25^ As for the yield, about 80% PC was obtained with both techniques. However, the protein concentration was much higher when ultrafiltration was used, as 13 mg/mL of PC was obtained with respect to 0.19 mg/mL of gel filtration.

**Table 1:** Comparison in PC Recovery after Gel Filtration and Ultrafiltration^a^

technique used	initial PC (mg/g_biomass_)	PC recovery (%)	PC concentration (mg/mL)	purity grade (Abs_620_/Abs_280_)
gel-filtration	98 ± 1.4	78 ± 8	0.19 ± 0.01	5 ± 0.2
ultrafiltration	98 ± 1.4	80 ± 7	13 ± 1.4^b^	5 ± 1

a PC was determined spectrophotometrically. Data shown are means ± S.D. of three independent experiments.

b *p* < 0.05 with respect to gel filtration.

### Total Carotenoid Extraction

Starting from the residual wet biomass after PC extraction, carotenoid extraction was performed by using the PLE technology. In order to compare the carotenoid extraction after PLE, a conventional acetone extraction was performed in parallel on dried raw biomass and on the dried residual biomass after PC extraction, as schematized in Figure 1. Usually, one of the mechanism employed to break cell wall is freeze-drying the biomass, which is a high energy-consuming treatment and causes rapid loss and degradation of carotenoids, thus affecting the bioactivity of the desired compounds.^26,27^ The results of the extractions are reported in Table 2. The carotenoid yield is expressed as mg of carotenoids extracted per g of dry biomass. It is interesting to notice that the conventional extraction allowed obtaining 62 mg of carotenoids from the raw biomass, whereas about 100 mg was recovered starting from the disrupted biomass. Thus, a significant increase (*p* < 0.05) in total carotenoid extraction was observed when the disrupted biomass was used. When PLE was employed on residual wet biomass, about 90 mg of carotenoids was obtained. Noteworthy, although the PLE did not increase the extraction yield with respect to the conventional method, the time needed to obtain carotenoids significantly decreased from 24 h to 30 min. In addition, no organic solvents were used, thus suggesting that this technology is green and very effective. In terms of total carotenoid content, the high-pressure procedure allowed obtaining a purer extract. In fact, as shown in Table 2, the conventional extraction allowed an increase in the carotenoid content up to 63% when the disrupted biomass was used instead of the raw one (*p* < 0.05). Surprisingly, PLE allowed a further increase of 250% in the total carotenoid content when compared to the conventional extraction technique on the disrupted biomass (*p* < 0.005) and 400% on the raw biomass (*p* < 0.05).

**Table 2:** Comparison between Conventional Extraction Performed on Raw Biomass and Biomass Post French Press Extraction and PLE Extraction after French Press in Terms of Extracted Carotenoids^a^

sample	carotenoid yield (mg/g_biomass_)	carotenoid content (mg/g_extract_)	zeaxanthin (mg/g_extract_)	β-carotene (mg/g_extract_)
raw biomass	62 ± 2	222 ± 24	2.7 ± 0.3	22 ± 4
post French press (conventional extraction)	100 ± 5^b^	362 ± 24^b^	33.4 ± 3.7^b^	320 ± 76^b^
post French press (PLE)	89 ± 6	911 ± 23^b^^,^^c^	48±5^b^^,^^d^	436 ± 60^e^

a Data shown are means ± S.D. of three independent experiments.

b *p* < 0.05 with respect to raw biomass.

c *p* < 0.005 with respect to conventional extraction after PC recovery.

d *p* < 0.05 with respect to conventional extraction after PC recovery.

e *p* < 0.005 with respect to raw biomass.

### Carotenoid Characterization by HPLC–DAD–MS

**Figure 2:**
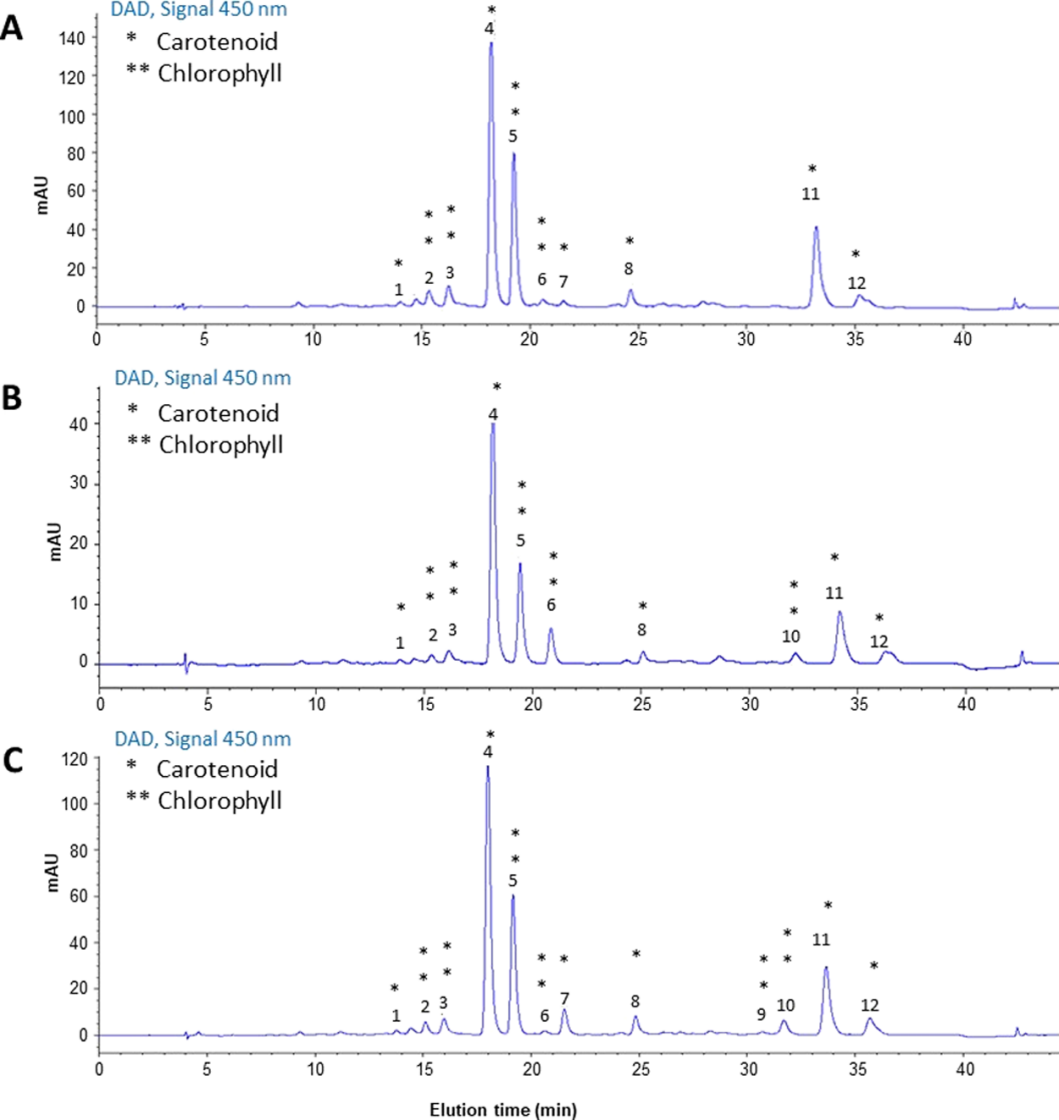
Representative HPLC–DAD chromatograms of carotenoids extracted from *G. phlegrea*. (A) Conventional extraction of raw biomass; (B) conventional extraction of the residual biomass after PC extraction; (C) PLE extraction of the residual biomass after PC extraction. * indicates carotenoids and ** indicates chlorophylls. Peak numbers and their identification are reported in Table 3.

Carotenoids obtained by the PLE technique were analyzed by high-performance liquid chromatography coupled to the diode array detector and mass spectrometry detector (HPLC–DAD–MS) in order to collect more information about the specific pigments (carotenoids and chlorophylls). When possible, a tentative identification was accomplished by combining the information provided by UV–vis spectra from DAD, [M + H]^+^, and MS/MS fragmentation patterns from the mass spectrometry detector and bibliographic search (Table 3). Chromatographic profiles shown in Figure 2 revealed that the extract obtained by PLE with ethanol is the one with the highest number of pigments.

**Table 3:** Pigments Detected in *G. phlegrea* Extracts

peak	identification	RT (min)	UV–vis max, nm	[M + H]^+^*m*/*z*	MS/MS main fragments detected
1	carotenoid	13.706	450, 475	664.3	607.5, 551.5, 495.4
2	hydroxychlorophyll *a*	15.062	430, 663	910.1	893.0, 631.8, 614.5
3	chlorophyll-type	15.696	426, 665	940.7	629.4, 661.4, 907.7, 852.7, 574.4
4	zeaxanthin^a^	17.926	428, 450,476	569.6	551.5
5	chlorophyll *a*^a^	19.092	432, 664	894.0	615.4, 583.3
6	chlorophyll *a*′	20.523	430, 665	894.1	615.6
7	carotenoid	21.453	445, 471		
8	carotenoid	24.772	450, 476	584.7	564.8
9	pheophytin *a*	30.425	408, 666	872.1	594.0, 683.3, 535.5
10	pheophytin *a*′	31.681	408, 666	871.9	593.8
11	β-carotene^a^	33.685	450, 475	537.7	
12	carotenoid	35.591	446, 472	592.8	533.4

a Identification corroborated by comparison with commercial standards; RT: retention time.

Peaks numbers 4, 5, and 11 stood out as the most relevant ones, and they could be tentatively identified as zeaxanthin, chlorophyll *a*, and β-carotene, respectively. These pigments were also present in the pressurized liquid extracts obtained with ethanol from other microalgae (*Neochloris oleoabundans*^20^ and *Porphyridium cruentum*(21)). Protonated ions of these compounds were detected (*m*/*z* 569.6 [M + H]^+^ for zeaxanthin, *m*/*z* 894.0 [M + H]^+^ for chlorophyll *a* and *m*/*z* 537.7 [M + H]^+^ for β-carotene), along with fragment ions of zeaxanthin and chlorophyll *a* produced by the loss of a water molecule (*m*/*z* 551.5 [M + H – H_2_O]^+^) or phytyl group (*m*/*z* 615.4 [M + H – C_20_H_38_]^+^), respectively. Furthermore, the identification of these three compounds was corroborated by the injection of commercial standards.

Other minor chlorophylls, peaks 2 and 9 were tentatively assigned as hydroxychlorophyll *a*(20) and pheophytin *a*(11) because of their UV–vis UV and MS/MS spectra, showing the particular loss of a phytyl group. Peaks number 6 and 10 have been tentatively identified as chlorophyll *a*′ and pheophytin *a*′ in concordance with their spectra, similar to those of chlorophyll *a* and pheophytin *a* but presenting longer retention times. Peak number 3 presented the characteristic absorbance spectrum of chlorophylls and therefore have been designed as chlorophyll-type.

The rest of the minor peaks in the chromatogram presented the characteristic absorbance spectrum of carotenoids. With the exception of peak number 7, that could not be detected in MS due to the lack of enough ionization efficiency, the rest of carotenoids were characterized in terms of [M + H]^+^, and many fragments from MS/MS were detected. However, a tentative identification was not possible. On the other hand, it is not the first time that peak number 12 has been reported. This carotenoid with the UV–vis spectrum with maximums at 446 and 472 nm was previously mentioned in gas expanded liquid extracts obtained with 75% of ethanol from the microalga *Scenedesmus obliquus*.^28^ In conclusion, the pigment analysis revealed β-carotene and zeaxanthin as the two main carotenoids in all extracts in agreement with Marquardt,^29^ but with a different *Galdieria* species (*G. sulphuraria*).

In addition, a method based on HPLC–DAD was employed to quantify the amount of zeaxanthin and β-carotene. To fit the calibration curves prepared with the commercial standards of both pigments, the samples analyzed were diluted in pure methanol at different concentrations: 10 mg/mL for the conventional extraction starting from raw biomass and 1 mg/mL for the two extracts obtained after French press. Quantification results are reported in Table 2. As expected, PLE improved the amount of both pigments. Moreover, the increase obtained was surprisingly interesting: up to 40% in comparison with the ones obtained by conventional extraction and to about 2000 times with respect to the raw biomass.

### Total Lipid Extraction

To further improve the biorefinery design, after the PLE extraction a lipid extraction was carried out using supercritical CO_2_ (ScCO_2_). Notably, both PLE and SFE were performed on the same apparatus, without the need to recover the biomass from the extraction cell after carotenoid extraction. In particular, after PLE, CO_2_ was injected in the extraction cell to push out ethanol-containing carotenoids. Afterward, pressure was increased to the super critical point, and lipids were extracted (Figure S1). As a benchmark, conventional chloroform/methanol extraction was carried out on raw dried biomass and on the residual dried biomass after PC extraction. Results of the extractions are reported in Table 4. The ScCO_2_ extraction allowed obtaining the same amount of lipids that those by conventional extraction, avoiding the use of an organic solvent. This result was quite surprising, as the lipids extracted are the third class of molecules obtained in a biorefinery approach. When compared with our previous results,^14^ we found a lower recovery in lipid yield, but this could be due to a different extraction method used.

**Table 4:** Comparison between Conventional Extractions Performed on Raw Biomass and Biomass Post French Press Extraction and SFE Extraction after French Press in Terms of Extracted Lipids^a^

sample	lipid yield (mg/g_biomass_)
raw biomass (conventional extraction)	110 ± 3
post French press (conventional extraction)	164 ± 6^b^
post French press (SFE)	184 ± 5^b^

a Data shown are means ± S.D. of three independent experiment.

b *p* < 0.05.

### Evaluation of Biocompatibility and Antioxidant Activity of Lipophilic Extract Obtained by PLE Extraction on Eukaryotic Cells

**Figure 3:**
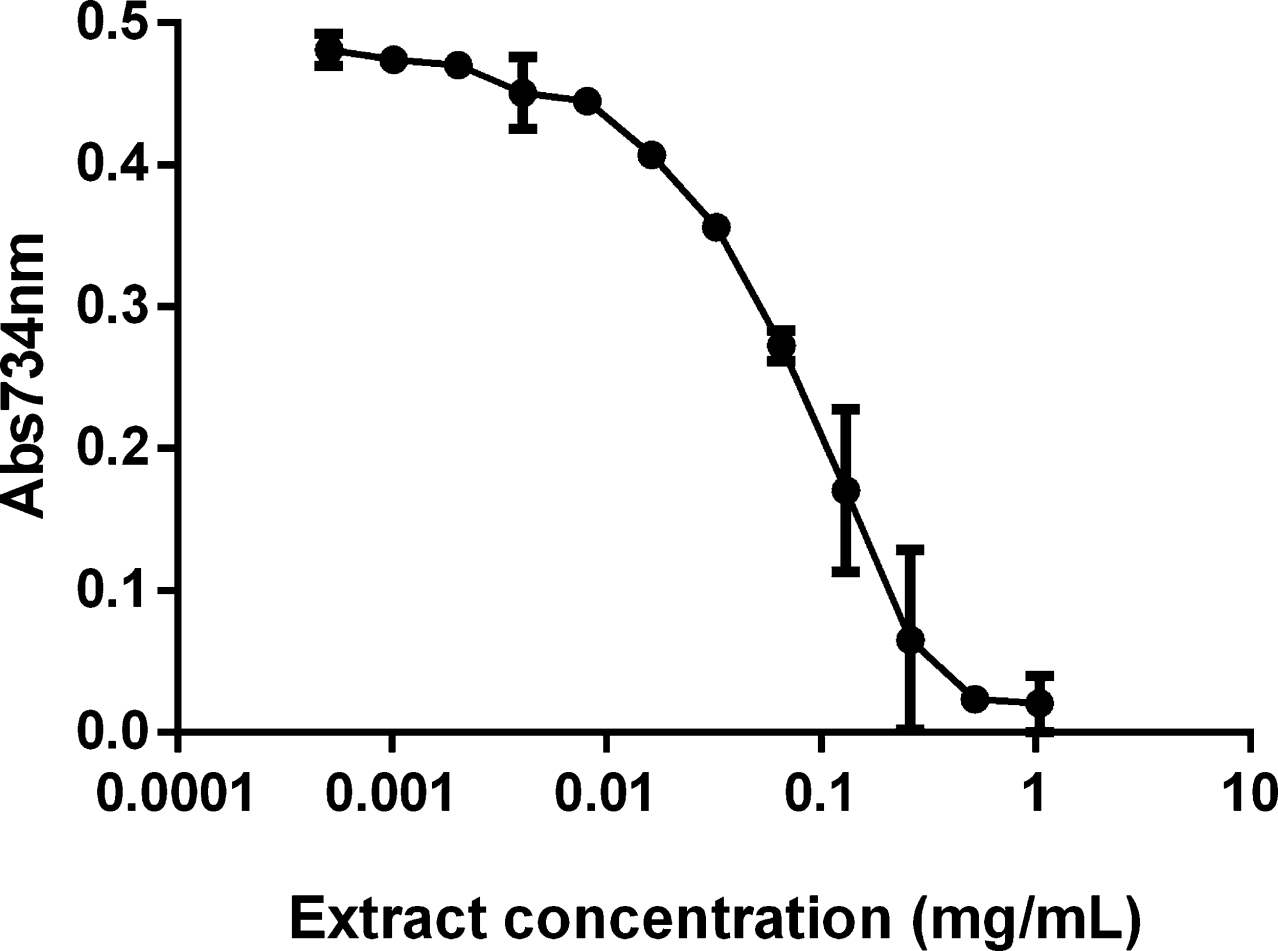
ABTS assay on carotenoids extracted from *G. phlegrea*. ABTS scavenging activity of different concentrations of the lipophilic extract (mg/mL) obtained by PLE from *G. phlegrea*. Data shown are means ± S.D. of three independent experiments.

**Figure 4:**
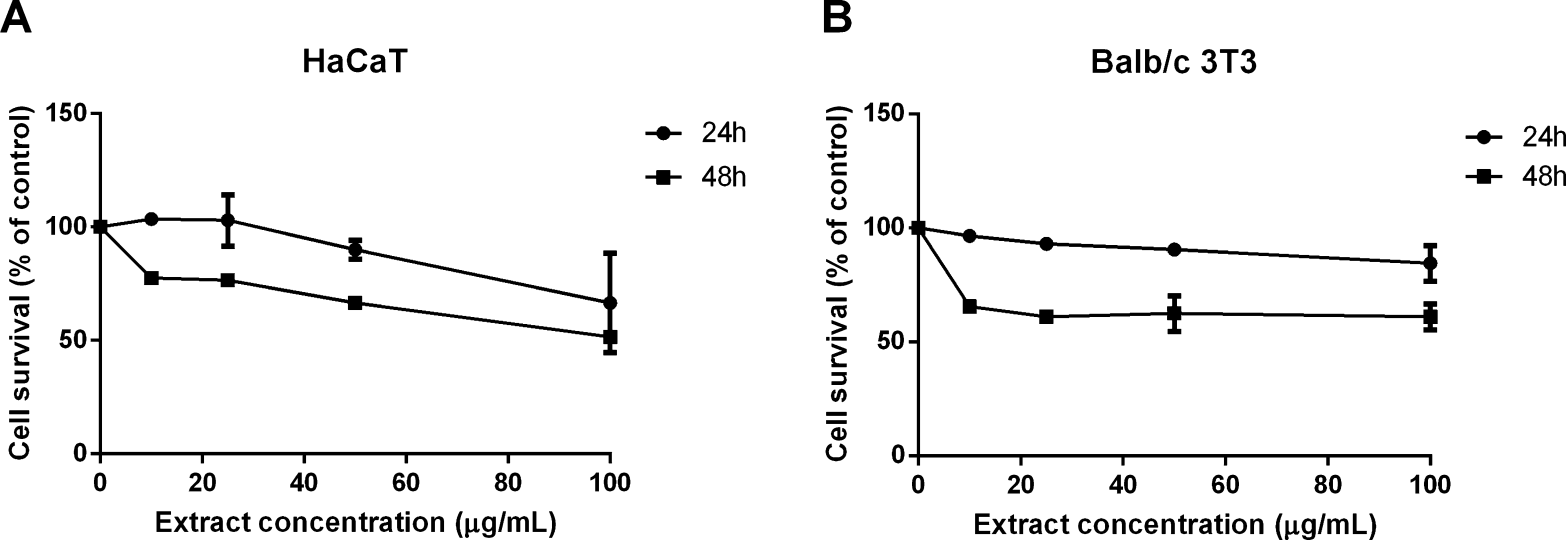
Effect of the lipophilic extract on the viability of HaCaT and BALB/c 3T3 cells. Dose–response curves of HaCaT (A) and BALB/c 3T3 (B) cells after 24 h (black circles) and 48 h (black squares) incubation with increasing concentrations of lipophilic extracts obtained by PLE (10–100 μg/mL). Cell viability was assessed by the MTT assay, and cell survival expressed as percentage of viable cells in the presence of the lipophilic extract under test, with respect to control cells grown in the absence of the extract. Data shown are means ± S.D. of three independent experiments.

To verify if the carotenoids extracted by the PLE technique were biologically active and safe for humans, their in vitro antioxidant activity, along with their biocompatibility on eukaryotic cells, was tested. The results of the in vitro ABTS colorimetric assay are shown in Figure 3 and clearly indicate that the lipophilic extract is endowed with a significant antioxidant activity. Its IC_50_ value, that is, the concentration of the extract that can inhibit 50% of the radical, is 50 μg/mL. This result is much lower than those reported in the literature, as the IC_50_ value here obtained is about 1600 times lower than others reported with different microalgae.^30^ The biocompatibility of the extract was tested by a time-course and dose–response test on immortalized murine fibroblasts (BALB/c 3T3) and immortalized human keratinocytes (HaCaT). Cell viability was assessed by the tetrazolium salt colorimetric (MTT) assay, and cell survival was expressed as the percentage of viable cells in the presence of the extract compared to that of control samples. As shown in Figure 4A,B, after 48 h, cell viability was not affected up to 50 μg/mL, while at the highest concentration tested (100 μg/mL), a 50% reduction of cell viability was observed.

### Protective Effect of the Lipophilic Extract against Oxidative Stress on HaCaT Cells

**Figure 5:**
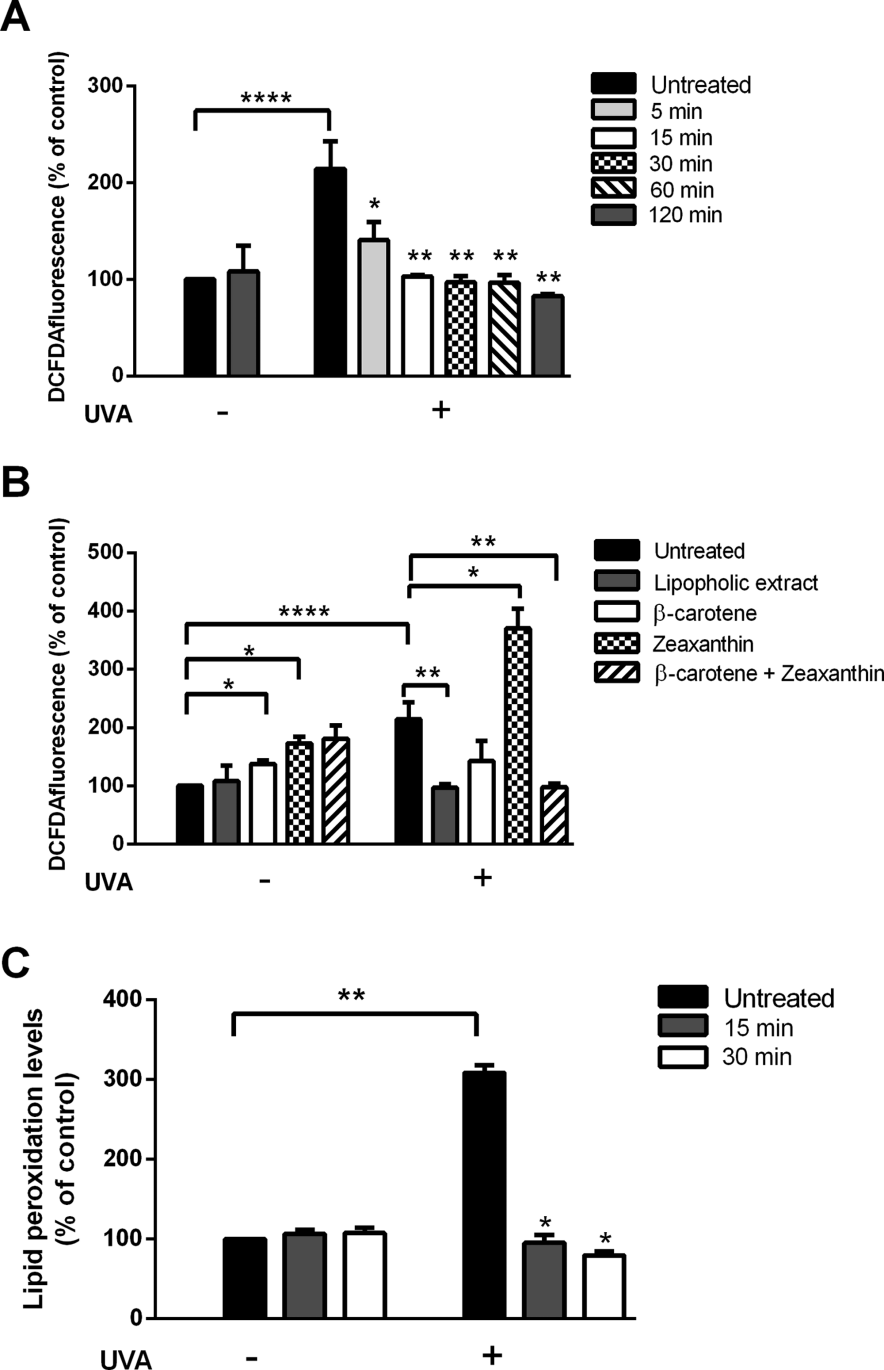
Antioxidant effect of the lipophilic extract from *G. phlegrea* on stressed HaCaT cells. Cells were preincubated in the presence of 50 μg/mL lipophilic extract from different lengths of time, prior to be irradiated by UVA (100 J/cm^2^). (A) Determination of intracellular ROS levels by DCFDA assay. Cells were incubated for 5 min (light grey bars), 15 min (white bars), 30 min (black-squared bars), 60 min (dashed bars), or 120 min (dark grey bars) with the lipophilic extract in the absence (−) or in the presence (+) of UVA. Black bars are referred to untreated cells. For each experimental condition, ROS production was measured and a percentage of the ratio between ROS production in treated cells and ROS production in untreated cells was calculated and reported in the graph. (B) Comparison of the protective effect of the lipophilic extract with commercial antioxidants by the DCFDA assay. Cells were incubated for 30 min prior to UVA exposure. Black bars are referred to untreated cells in the absence (−) or in the presence (+) of UVA. Grey bars are referred to cells incubated with 50 μg/mL lipophilic extract; white bars are referred to cells incubated with 24 μg/mL β-carotene; black squared bars are referred to cells incubated with 2.4 μg/mL zeaxanthin; and dashed bars are referred to cells incubated with both β-carotene and zeaxanthin. (C) Analysis of lipid peroxidation levels evaluated by TBARS assay. Cells were preincubated with the lipophilic extract for 15 (grey bars) or 30 min (white bars) before UVA irradiation. Values are expressed as % with respect to control (i.e. untreated) cells. For each experimental condition, lipid peroxidation levels were measured, and a percentage of the ratio between lipid peroxidation levels in treated cells and lipid peroxidation levels in untreated cells was calculated and reported in the graph. Data shown are means ± S.D. of three independent experiment. * indicates *p* < 0.05, ** indicates *p* < 0.005, and **** indicates *p* < 0.0001.

As the lipophilic extract obtained by PLE contains antioxidants, the potential protective effect against oxidative stress was analyzed on a cell-based model. As a cell system, we chose immortalized keratinocytes as they are normally present in the outermost layer of the skin and UVA radiations as a source of stress. Cells were treated with 50 μg/mL extracts for different lengths of time (from 5 to 120 min), and then oxidative stress was induced by UVA irradiation (100 J/cm^2^). Immediately after irradiation, ROS levels were measured by using H_2_DCF-DA as a probe. For each set of experiments, untreated cells were used as a control. Under physiological conditions (i.e., in the case of untreated cells), a physiological release of ROS is observed (100%). As shown in Figure 5A, no effect on ROS levels was observed when cells were incubated with the extract for 120 min (grey bars), whereas UVA treatment significantly increased DCF fluorescence intensity (black bars). Interestingly, pretreatment of cells with the lipophilic extract, prior to UVA exposure, resulted in an inhibition of ROS production, which was clear already after 5 min of pretreatment. We then performed a comparison between the antioxidant activity of the total lipophilic extract obtained by PLE and commercial β-carotene and zeaxanthin, the two most abundant species identified in the extract. On the basis of the quantification data reported in Table 2, we calculated that, when the lipophilic extract was tested at 50 μg/mL, the amount of β-carotene corresponded to 24 μg/mL and that of zeaxanthin to 2.4 μg/mL. Thus, HaCaT cells were preincubated for 30 min with either: 50 μg/mL of lipophilic extract; 24 μg/mL of β-carotene; 2.4 μg/mL of zeaxanthin; a mixture of both carotenoids. At the end of incubation, oxidative stress was induced as previously mentioned. Alteration of ROS levels was measured by using H_2_DCF-DA. As shown in Figure 5B, a significant increase in ROS production was observed when cells were incubated with commercial β-carotene (white bars) or zeaxanthin (black squared bars), also in the absence of any UVA exposure. Interestingly, only the mixture of both commercial carotenoids (dashed bars), as well as the lipophilic extract (grey bars), were able to counteract oxidative stress in a similar way. The protective effect of the lipophilic extract was also confirmed by analyzing the lipid peroxidation levels. To this purpose, TBARS were measured and related to lipid peroxidation levels. A significant increase in lipid peroxidation levels was observed after UVA treatment, but, notably, this effect was abolished when cells were pretreated with the lipophilic extract, either after 15 or 30 min preincubation (grey and white bars, respectively). Treatment of cells with the lipophilic extract did not alter significantly lipid peroxidation levels (Figure 5C).

### Nrf-2 Regulates the Antioxidant Activity of the Lipophilic Extract

**Figure 6:**
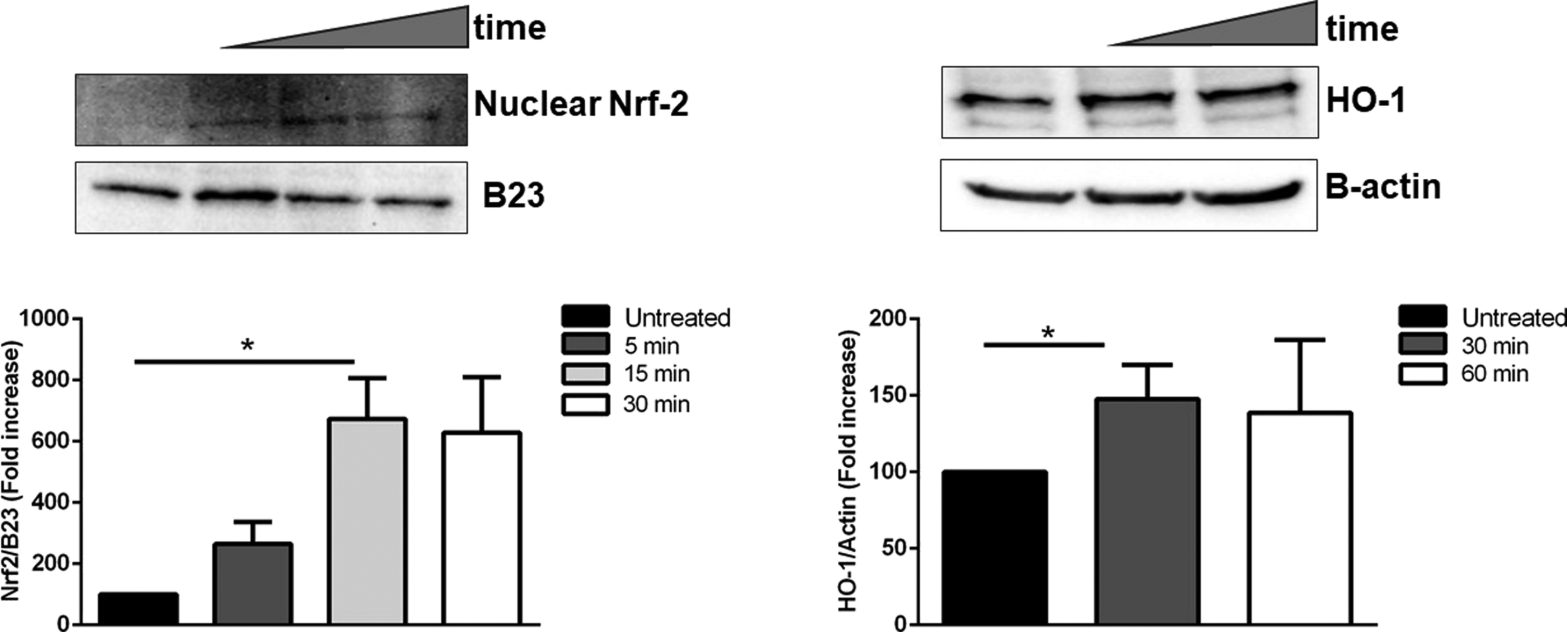
Effect of the lipophilic extract on Nrf-2 activation on HaCaT cells. Cells were incubated with 50 μg/mL lipophilic extract obtained by the PLE technique for different lengths of time and then nuclear (A) or cytosolic (B) proteins were analyzed by western blotting. (A) Western blot analysis of nuclear Nrf-2 after 5 min (dark grey bar), 15 min (light grey bar), and 30 min (white bar) incubation. Nuclear Nrf-2 and B23 were quantified by densitometric analysis. The ratio between Nrf-2 and B23 of each treated sample was then related to the ratio Nrf-2/B23 of untreated cells, considered as 100%. (B) Western blot analysis of cytosolic HO-1 was performed after incubation with 50 μg/mL of the extract for 30 min (dark grey bar) and 60 min (white bar). HO-1 and β-actin were quantified by densitometric analysis, and the ratio HO-1/β-actin of each treated sample was then related to the ratio HO-1/β-actin of untreated cells, considered as 100%. Data shown are means ± S.D. of three independent experiments. * indicates *p* < 0.05 with respect to control cells.

To understand the molecular mechanism responsible for the protective effect of the lipophilic extract, the involvement of the transcription factor Nrf-2 was analyzed. Under normal physiological conditions, Nrf-2 is associated with Keap-1, which retains Nrf-2 in the cytosol and directs it to the proteasomal degradation. Upon either oxidative stress induction and/or in the presence of antioxidants, Keap-1 dissociates from Nrf-2, which is translocated to the nucleus where it binds to antioxidant responsive elements sequences and activates the transcription of several phase-II detoxifying enzymes.^31^ Thus, we incubated HaCaT cells in the presence of the lipophilic extract for different length of time (from 5 to 30 min), and lysates were analyzed by western blot analysis, using Nrf-2 antibody. As shown in Figure 6A, an increase in nuclear Nrf-2 was observed after 15 min of incubation. The activation of Nrf-2 was further confirmed by analyzing the translation level of the heme oxygenase-1 (HO-1) by western blot analysis. HO-1 is a ubiquitous and redox-sensitive inducible stress protein that degrades heme to CO, iron, and biliverdin.^32^ The importance of this protein in physiological and pathological states is underlined by the versatility of HO-1 inducers and the protective effects attributed to heme oxygenase products in conditions that are associated with moderate or severe cellular stress. Thus, HaCaT cells were incubated for 30 and 60 min, and lysates were analyzed by western blot analysis, using a HO-1 antibody. As shown in Figure 6B, an increase in HO-1 levels was observed after 30 min of incubation.

## Conclusions

One of the aims of green chemistry is to preserve the natural environment, promoting a better use of resources and limiting the negative influence of human involvement, such as the use of procedures that require the use of toxic solvents.^33^ Compared to conventional extractions, this innovative green biorefinery approach is able to extract, in cascade, three different bioactive compounds from the microalga *G. phlegrea*. In combination, the described process allows achieving higher yields of PC, carotenoids, and lipids using Generally Recognized As Safe (GRAS) solvents, in shorter time and with less solvent consumption. Here, we demonstrated that PLE using ethanol has a high potential to extract carotenoids from *G. phlegrea*. Moreover, as *G. phlegrea* is an eukaryotic microalga, it possesses a robust cell wall, which prevents the release of intracellular products. The idea of breaking the biomass by high pressure homogenization allowed to isolate PC and helped the subsequent release of carotenoids. Both final products, PC, and carotenoids were biologically active in terms of antioxidant activity.^14^ These results will open the way to the idea of commercializing carotenoids from microalgae for cosmeceutical applications. In conclusion, this work will help to achieve a complete valorization of the *G. phlegrea* microalga biomass. The results can then contribute to increase the revenue streams of the process, in order to compensate the large cultivation and downstream cost for biomass production and, finally, turn positive the economic balance of the microalgae biorefinery. Furthermore, they contribute to develop a green process which can also increase the social acceptance of industrial microalgal products.

## Supporting Information Available

Schematic representation of the equipment employed for compressed fluid extractions (PDF)
