# Supplementary material for: Green Compressed Fluid Technologies To Extract Antioxidants and Lipids from Galdieria phlegrea in a Biorefinery Approach
Source: ACS Sustain Chem Eng. 2020 Jan 31;8(7):2939–47. doi: 10.1021/acssuschemeng.9b07505 (PMC8016174; doi:10.1021/acssuschemeng.9b07505)
Supplement: Supplementary file 1 — sc9b07505_si_001.pdf [file sc9b07505_si_001.pdf]

SUPPORTING INFORMATION

Green compressed fluid technologies to extract antioxidants and lipids from *Galdieria phlegrea* in a biorefinery approach

Paola Imbimbo[a], Monica Bueno[b], Luigi D'Elia[a], Antonino Pollio[c], Elena Ibañez[b], Giuseppe Olivieri[d,e*], Daria Maria Monti[a*]

[a] Department of Chemical Sciences, University of Naples Federico II, via Cinthia 4, 80126, Naples, Italy

[b] Laboratory of Foodomics, Institute of Food Science Research, CIAL, CSIC, Nicolás Cabrera 9, 28049 Madrid, Spain.

[c] Department of Biology, University of Naples Federico II, via Cinthia 4, 80126, Naples, Italy

[d] Bioprocess Engineering Group, Wageningen University and Research, Droevendaalsesteeg 1, 6700AA, Wageningen, the Netherlands

[e] Department of Chemical, Materials and Industrial Engineering, University of Naples Federico II, Piazzale Tecchio 80, 80125, Napoli, Italy

*Corresponding Authors: G.O. giuseppe.olivieri@wur.nl; D.M.M. mdmonti@unina.it

Number of pages: 2

Number of figures: 1

Number of tables: 0

1. Compressed Fluid Extraction apparatus..2

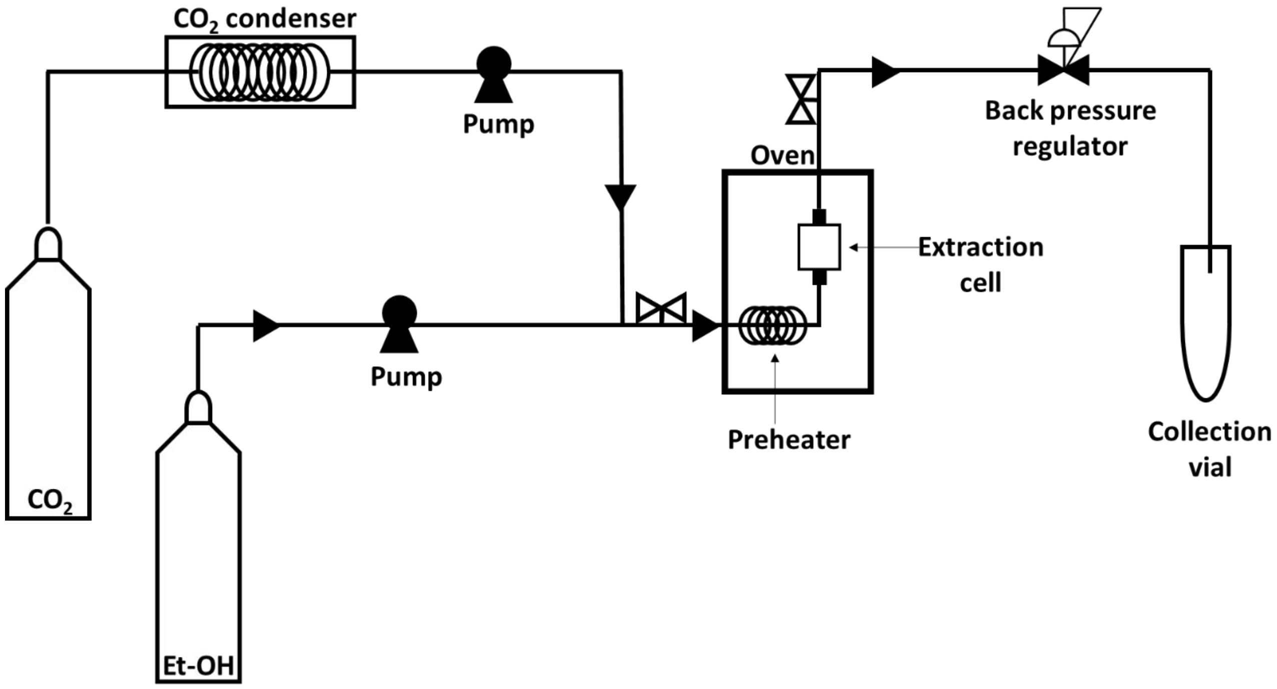

Figure S1: Compressed Fluid Extraction apparatus. The oven is equipped with a preheater and an extraction cell. Two pumps are connected to the extraction cell and a back pressure regulator connects the oven to the collection vial.
